# New Web-Based System for Recording Public Health Nursing Practices and Determining Best Practices: Protocol of an Exploratory Sequential Design

**DOI:** 10.2196/45342

**Published:** 2023-06-12

**Authors:** Kyoko Yoshioka-Maeda, Hiroshige Matsumoto, Chikako Honda, Misa Shiomi, Kazuya Taira, Noriko Hosoya, Miki Sato, Yuka Sumikawa, Hitoshi Fujii, Takahiro Miura

**Affiliations:** 1 Department of Community Health Nursing Division of Health Sciences & Nursing, Graduate School of Medicine The University of Tokyo Bunkyo-ku Japan; 2 Department of Innovative Public Health Nursing Graduate School of Medicine Kyoto University Kyoto City Japan; 3 Department of Nursing, Faculty of Healthcare Sciences Chiba Prefectural University of Health Sciences Chiba City Japan; 4 Department of Health Promotion National Institute of Public Health Wako City Japan; 5 Department of Gerontological Homecare & Long-term Care Nursing, Division of Health Sciences & Nursing The University of Tokyo Bunkyo-ku Japan; 6 Department of Medical Statistics, School of Nursing Mejiro University Saitama City Japan; 7 Human Augmentation Research Center National Institute of Advanced Industrial Science and Technology Kashiwa City Japan

**Keywords:** community-based activity, evidence-based practice, individual care, information and communication technology, program development, public health nursing, quality assurance, digitalization, eHealth, electronic record

## Abstract

**Background:**

Digitalization and information and communication technology (ICT) promote effective, efficient individual and community care. Clinical terminology or taxonomy and its framework visualize individual patients’ and nursing interventions’ classifications to improve their outcomes and care quality. Public health nurses (PHNs) provide lifelong individual care and community-based activities while developing projects to promote community health. The linkage between these practices and clinical assessment remains tacit. Owing to Japan’s lagging digitalization, supervisory PHNs face difficulties in monitoring each department’s activities and staff members’ performances and competencies. Randomly selected prefectural or municipal PHNs collect data on daily activities and required hours every 3 years. No study has adopted these data for public health nursing care management. PHNs need ICTs to manage their work and improve care quality; it may help identify health needs and suggest best public health nursing practices.

**Objective:**

We aim to develop and validate an electronic recording and management system for evaluating different public health nursing practice needs, including individual care, community-based activities, and project development, and for determining their best practices.

**Methods:**

We used a 2-phase exploratory sequential design (in Japan) comprising 2 phases. In phase 1, we developed the system’s architectural framework and a hypothetical algorithm to determine the need for practice review through a literature review and a panel discussion. We designed a cloud-based practice recording system, including a daily record system and a termly review system. The panels included 3 supervisors who were prior PHNs at the prefectural or municipal government, and 1 was the executive director of the Japanese Nursing Association. The panels agreed that the draft architectural framework and hypothetical algorithm were reasonable. The system was not linked to electronic nursing records to protect patient privacy. Phase 2 validated each item through interviews with supervisory PHNs using a web-based meeting system. A nationwide survey was distributed to supervisory and midcareer PHNs across local governments.

**Results:**

This study was funded in March 2022 and approved by all ethics review boards from July to September and November 2022. Data collection was completed in January 2023. Five PHNs participated in the interviews. In the nationwide survey, responses were obtained from 177 local governments of supervisory PHNs and 196 midcareer ones.

**Conclusions:**

This study will reveal PHNs’ tacit knowledge about their practices, assess needs for different approaches, and determine best practices. Additionally, this study will promote ICT-based practices in public health nursing. The system will enable PHNs to record their daily activities and share them with their supervisors to reflect on and improve their performance, and the quality of care to promote health equity in community settings. The system will support supervisory PHNs in creating performance benchmarks for their staff and departments to promote evidence-based human resource development and management.

**Trial Registration:**

UMIN-ICDR UMIN000049411; https://tinyurl.com/yfvxscfm

**International Registered Report Identifier (IRRID):**

DERR1-10.2196/45342

## Introduction

### Background

Digital technologies promote innovation in health care and nursing [1]. Notably, a previous study categorized different applications of information and communication technology (ICT) in nursing practice into assessment, clinical intervention, health promotion, and service organization [2]. Regarding assessment, nurses have used electronic clinical recording systems [3] to assist in clinical decision-making for individual care [4], meet patients’ health needs [5], and reduce overwork time and medical care expenditure [6]. Meanwhile, ICT-based health promotion programs can improve community health [7]. ICT also ensures efficient staffing and supports health workers’ decision-making, communication, training, and health system management [1]. Therefore, digitalization and ICT promote effective and efficient care for individuals and communities. However, previous research has primarily focused on hospital- or clinic-based individual care [8]; little work has been done on the function of ICT in public health nursing [2].

Clinical terminology or taxonomy and its framework help visualize the classification of patients and nursing practice and improve health equity in multidisciplinary settings [9-14]. Nursing practices are based on nursing diagnosis systems [11] and interventions [12]. Focusing on patient outcomes is essential to improve nursing care and management quality [13,14]. However, while prior studies examine individual patients, knowledge about public health nursing is scarce. Under each country's different cultures, policies, and health care systems, public health nurses (PHNs) provide a wide range of lifelong care, support at the group level, and community-based activities. They also create policies, programs, projects, and health care systems that meet the needs of each community [15]. Individual care takes up most of a PHN’s time [16], making it difficult to perform community-based activities and assessments [17], which are the basis of developing needs-oriented projects. Although these projects are vital for improving health equity in the community [18], evaluating the necessity of these practices and their linkage depends on the PHN’s tacit knowledge. Therefore, in this field, assessment items core to clinical reasoning considering sociocultural backgrounds are required.

Given the rapidly changing health care needs that affect services, systems, and staffing [8], digitalization is still recent, especially in PHN [19]. The COVID-19 pandemic has accelerated the shortage of PHNs and digitalization in Japan [20]. Approximately 70% of PHNs in Japan work as prefectural or municipal public servants [21] and work in various departments. As Japan has the world’s oldest population and the lowest birth rate, the national government must encourage digitalization and reduce the number of civil servants by half by 2040 [22]. Therefore, the total number of PHNs will potentially be halved by 2040. To ensure the quality of public health nursing, Japan has a national license system for PHNs [23]. Additionally, supervisory PHNs must manage practices in each department [24] while assessing the workloads of their staff and developing their competencies. Owing to the lack of ICT systems, supervisory PHNs may be unable to understand the happenings in each department and the performance and competencies of each staff member. An ICT system is needed to support their management and human resource development.

Furthermore, electronic record systems collect data on nursing care and promote evidence-based management in hospitals [6,10-13,25,26]. The Omaha system also enhances such management for each patient in community settings [14]. In Japan, the national government conducts a public health nursing activities survey every 3 years [27]. To this end, randomly selected prefectural or municipal PHNs gather data on their daily activities and the required hours [27]. However, no study has applied these data to elucidate public health nursing care management. To encourage evidence-based practices and nursing management, PHNs require an electronic recording and management system that identifies health needs and suggests the best public health nursing practices. The system would help PHNs think about their daily activities and share information with their supervisors to improve the quality of care and competencies, thus ensuring health equity in the community.

### Objective

This study will develop and validate an electronic recording and management system to evaluate the necessity of different public health nursing practices—including individual care, community-based activities, and project development—and to determine their best practices.

## Methods

### Study Design

This study used an exploratory sequential design, which was conducted in Japan [28]. This design was suitable for exploring unknown questions using qualitative methods and developing a new instrument that can be validated by quantitative research analysis [28]. This study has 2 phases. Phase 1 will include a panel discussion to develop a structure for the electronic recording and management system. Phase 2 will confirm the validity of the proposed items through interviews with expert PHNs and a nationwide survey of PHNs.

### Participants

The research team worked in public health nursing, home care nursing, public health, statistics, and computer sciences. This study is divided 2 phases. In phase 1, the research team conducted a literature review and a panel discussion that included 3 supervisors with experience working as prefectural or municipal government PHNs and 1 executive director of the Japan Nurse Association. The criteria for convening the expert panels meeting were to gather their opinions on the draft architectural framework—a hypothetical algorithm to determine the need for practice review; moreover, each item was developed by the research team.

In phase 2, the participants of interviews will be supervisory PHNs in prefectures or municipalities. Additionally, to ensure nationwide representation, we will include supervisory PHNs and midcareer PHNs with experience working in all local governments in Japan except those that completed the interviews. The inclusion criteria of midcareer PHNs have their cases and experience working for 6 to 20 years. The exclusion criteria for them are a managerial position.

### Recruitment

In phase 1, the research team recruited a panel of experts using snowball sampling. In phase 2, they recruited 5 to 10 supervisory PHNs as participants, who worked in prefectures or municipalities to avoid bias in the functions and roles of public health centers. Before starting the nationwide survey, we sent letters, including the study description, to supervisory PHNs. We used a mailing list of all supervisory PHNs from the Ministry of Health, Labour and Welfare to announce the survey by email. We expect approximately 530 local governments to participate based on a collection rate of 30% in a previous study that used a similar survey methodology [29].

### Procedures

#### Phase 1: Develop an Architectural Framework

**Figure 1:**
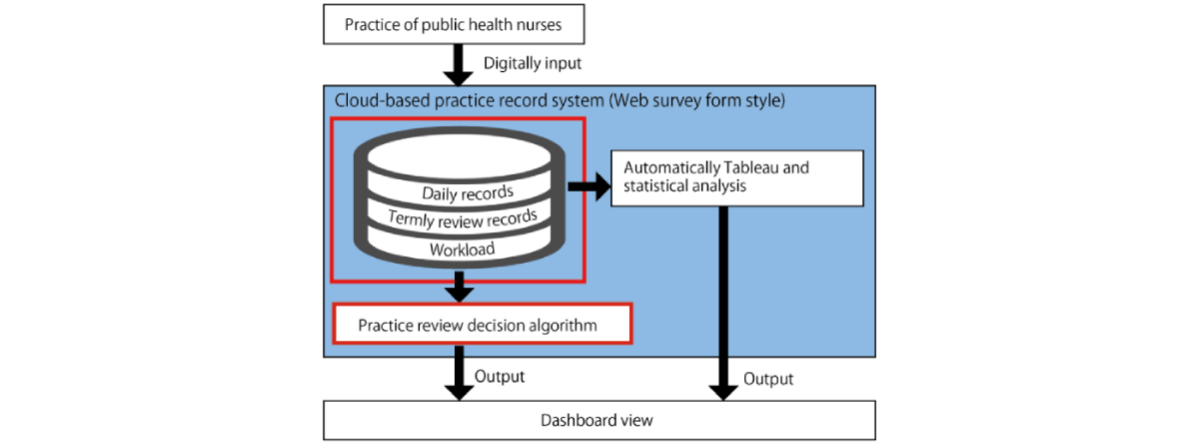
An architectural framework for an electric recording and management system.

**Figure 2:**
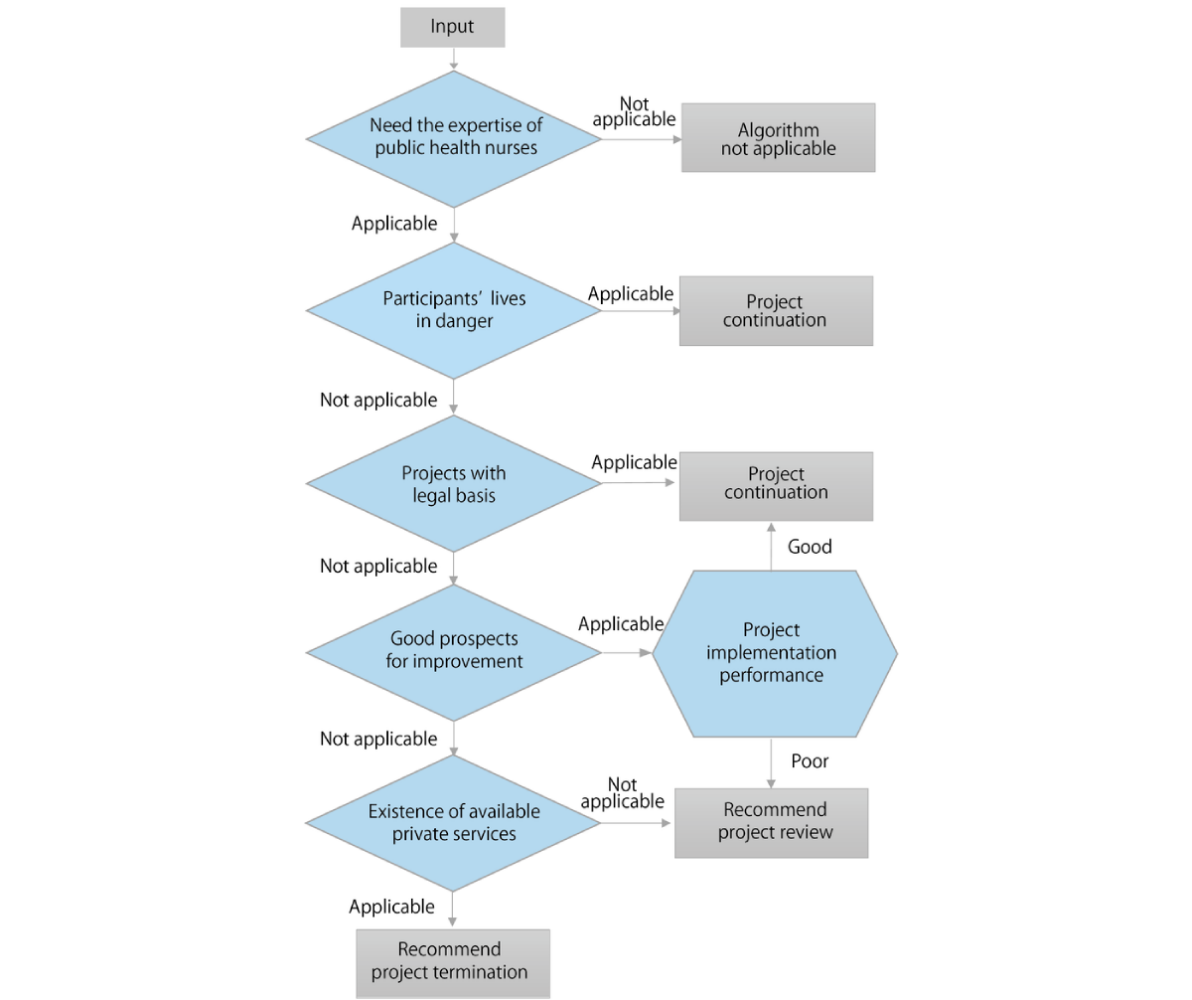
A hypothetical algorithm for determining the need for practice review.

Based on a literature review [6,25-27,30-32] and discussions at 10 meetings, the research team created an architectural framework (Figure 1) for an electronic recording and management system. This study reports the construction of the system in the blue background area, especially the development of the system input items, and the hypothetical algorithm based on the practical experiences in the red frame areas. The data will be stored on a server contracted by the research team. The system can be accessed via a web browser on a touchscreen device or personal computer. We will collect data through a web-based survey form and a cloud-based practice recording system. Statistical analysis will be performed based on the data, and the results will be presented on the dashboard view as tables or graphs. Figure 2 shows the hypothetical algorithm to determine the need for practice review. Based on each PHN’s assessment regarding individual care, community activities, and project implementation, an algorithm will suggest appropriate practices and directions for improvement.

#### Phase 2: Confirming the Validity of the Proposed Items

In phase 2, the validity of the proposed items will be tested using interviews with expert PHNs and a nationwide questionnaire survey of supervisory and midcareer PHNs.

The semistructured interviews with supervisory PHNs will be conducted over a web-based meeting system. We will confirm that the validity of the survey items comprising each unit constructed in phase 2 is relevant to the cases handled by PHNs (ie, to verify that the PHNs will be able to answer the question). We will send the survey items to the interviewees in advance, including the items comprising the unit constructed in phase 1 and a semistructured interview guide. To protect the interviewees’ privacy, we will not record the interviews. To ensure the trustworthiness of the qualitative phase and to protect the interviewees’ privacy, 2 researchers will conduct the semistructured interviews in pairs, one acting as an interviewer and the other taking notes. After the interviews, we will send a memo of the content to the interviewees over email for confirmation. Based on the results, we will revise each item in the system and finalize it.

The nationwide survey will be conducted using a web-based form, and a URL or QR code for the survey will be distributed to supervisory and midcareer PHNs. We will ask the research participants to (1) select one highly necessary and one less necessary element of individual care, community-based activities, and project development that they have performed within the last month; and (2) enter select cases into our system and summarize them. The level of necessity of each activity will be measured using a 10-point Likert scale from 1 (very unnecessary) to 10 (highly necessary). Additionally, we will ask participants about the importance and incomprehensibility of each item and whether to include any additional items. Responses will be sent directly to the researcher (ie, they will not go through the supervisory PHNs).

### Data Analysis

In the qualitative phase, 3 researchers will read the interview transcripts (confirmed by the interviewees) and classify the contents in terms of the survey items using the content analysis method [33]. We will combine expressions with similar meanings into a single concept, whereas contradictory data will be left as is. After consulting with the project’s supervisory PHNs, we will revise the survey items. The items for the nationwide survey will be finalized after all the project members confirm their validity.

The nationwide survey will be conducted through a descriptive analysis of the components of the system and the subjective necessity of PHN practices. In this survey, PHNs will answer based on 2 types of activities: (1) highly unnecessary and (2) highly essential. We want to compare these 2 categories and confirm whether the items will effectively work to help identify the differences between each activity in terms of necessity. We will use Cronbach α to measure the degree of agreement between the importance calculated when the responses were applied to the system developed in phase 1 and the subjective importance expressed by PHN interviewees. We will also perform a decision tree or random forest analysis with the PHN’s subjective sense of significance as the outcome and the system configuration items as explanatory variables. The model with the highest prediction accuracy will be compared with the system created in phase 1. Finally, the panels will verify the nationwide survey results to confirm the external validity and consider whether the system may be improved based on the findings.

### Ethical Considerations

This study will be conducted following the Declaration of Helsinki. Ethical approval for this study was obtained from the Research Ethics Committee of the Faculty of Medicine of the University of Tokyo, review number ID=2022114NI, 2022114NI-(1), 2022114NI-(2). We will explain the study’s purpose and methods to the interviewees and advise them that their privacy will be protected in written form. Informed consent will be obtained from each participant. For the nationwide survey, only those who agree to participate in the study will be asked to respond to the questionnaire.

## Results

### Overview

This study was supported by the Ministry of Health, Labour and Welfare Japan research grant, which was acquired in April 2022. Interviews for supervisory PHNs were started in September 2022. Additionally, participants in the nationwide survey will be recruited in December 2022. The estimated date of the nationwide survey’s completion will be January 2023. The preliminary results will be submitted for publication in December 2023.

### Items From a Cloud-Based Practice Record System

In phase 1, the panels agreed that the draft architectural framework and a hypothetical algorithm were reasonable. After meetings with them, we decided that the system should not be linked to electronic nursing records to protect the privacy of each individual case. Items from a cloud-based practice record system are depicted in Tables 1 and 2. The items were divided into 2 domains. The first was a daily record system regarding individual care, community activities, and developing needs-oriented projects, whereas the second was their termly review system. Based on the Omaha system framework [14], we added assessment items from the perspectives of the individual, the family, and the environment, especially for individual care. We used 9 categories regarding a national survey of public health nursing activities [27]: infectious disease, intractable disease, disability, mental health, maternal and child health, health promotion, aging and long-term care, occupational health, and child welfare.

After developing the parts of the system, the researchers entered the data using typical vignette cases based on the national examination level and modified the items based on the results. The expert panels also filled in the items. However, the vignette cases were more straightforward than the actual clients. Therefore, we will ask PHNs to answer each item in the nationwide survey based on their actual cases (see below for more information).

**Table 1:** Items from a cloud-based practice record system: individual care.

System, unit, category/domain, and examples of items							Items, n
**Daily record system (N=71-89^a^)**							
	**Transdomain items (n=69)**						
		**Demographics of the PHNs^b^**				5	
			PHN ID				
			Dates				
			Districts				
			Activity domains				
			Time consumed				
		**Necessity**				44	
			**Individual**				
				Symptom: acute deterioration of medical condition			
				ADL^c^: dependent in basic ADLs			
				Acceptance: refusal of support, consultation, or treatment			
				Treatment: need medical care at home			
				Communication: difficulty in communication			
				Social support: absence of key persons			
				Emergency: risk of self-injury			
			**Family**				
				Symptom: family member’s poor health			
				Acceptance: family member’s refusal of support, consultation, or treatment			
				Communication: family member’s lack of problem awareness and coping skills			
				Social support: family member’s absence of someone to consult			
				Emergency: domestic violence			
			**Environment**				
				Housing: unsanitary living environment			
				Social resource: lack of social resources available			
				Economic: economic deprivation (insufficient income)			
				Neighborhood: trouble with neighbors			
		Daily review: the problem is worsening or is expected to worsen				9	
		Activity selection: cooperation of community residents is necessary to solve the problem				6	
		Task load: how irritated, stressed, and annoyed did you feel during the task?				5	
	**Domain-specific items (N=2-20^a^)**						
		**Necessity**					
			Infectious disease: risk of outbreak		10		
			Intractable disease: support immediately after diagnosis or discharge		5		
			Disability: problems in employment or social participation		5		
			Mental health: disturbance of life rhythm		2		
			Maternal and child health: caregiver’s risk in life history (eg, history of childhood abuse)		5		
			Health promotion: refusal of specific health guidance		7		
			Aging and long-term care: behavioral and psychological symptoms of dementia		4		
			Occupational health: young caregiver		20		
**Termly review system (N=14)**							
	Termly review: imbalance in the number and load of cases per PHN/district					4	
	Activity selection: accumulation of cases with similar needs					10	

^a^Number of items depends on the domain.

^b^PHN: public health nurse.

^c^ADL: activities of daily living.

**Table 2:** Items from a cloud-based practice record system: community-based activities and project development.

Unit, category/domain, and example of items							Items, n
**Daily record system (N=26)**							
	**Demographics of the PHNs^a^**						5
			PHNs ID				
			Dates				
			Districts				
			Activity domains				
			Time consumed				
	Necessity: risk to the life and well-being of the participants						4
	Daily review: fewer participants in the activity/project than expected						3
	Activity selection: people who have interrupted their participation and need individual follow-up						2
	Task load: decreased involvement of residents in the operation of activity/project						12
**Termly review system (N=57)**							
	Necessity: data available that support the necessity						12
	**Termly review**						
		Prediction and evaluation: initial health issue resolved and low probability of relapse				10	
		Disparity: inequity in service provision between regions or target groups				3	
		Substitutability: alternative private services available				5	
		Institutional issue: end of the year as a subsidized project				2	
		Resource: lack of cooperation with key persons in the district				8	
		Aggregated: long-term and large number of PHN inputs determined the project is less necessary				3	
	**Activity selection**						
		Community-based activities to project development: enactment or amendment of laws related to activities/project			10		
		Project to community-based activities: necessity to modify the operation of the project depending on the district			4		

^a^PHN: Public health nurse.

#### Daily Record System

The daily record system has 5 units: (1) demographic information of PHNs, (2) assessment of the necessity, (3) daily review, (4) activity selection, and (5) task load [34]. PHNs will enter related data daily, as described below.

1. Demographics of the PHN unit. PHNs will input their IDs, dates of their activities, districts, activity domains, and the time required for each activity.
2. Assessment of the necessity unit. The “necessity unit” determines how necessary each PHN activity is. The necessity of an individual case is determined by checking an applicable option (applicable, not applicable, or unknown) for each of the necessity judgment items in the domain to which each case corresponds to determine clinical urgency and any problems. The level of necessity of community-based activities and project development practices will be determined by checking the transdomain items and recording the number of people—the activity and practice targets.
3. Daily review unit. It evaluates PHN activities to determine whether they are appropriate and worth continuing. Items are distributed across the individual care, community-based activity, and project development subsets.
4. Activity selection unit. It examines movements across the different types of PHN activities. Individual care practices include items that speak to or can develop into community-based activities and project development practices.
5. Task load unit. It determines the difficulty level of each activity. Individual care difficulty will be measured using the Japanese version of the National Aeronautics and Space Administration task load index. This considers mental, physical, and temporal demands, level of effort, performance, and frustration [34].

#### Termly Review System

The termly review system assesses each activity by collecting monthly or semiannual daily reports and inputting additional items, including (1) necessity, (2) the termly review, and (3) activity selection units.

1. Necessity unit. It consists of items that determine the necessity of community-based activities and project development from a long-term perspective. The term review unit in individual care activities is automatically checked based on accumulated daily reports.
2. Termly review unit. The termly review items for community-based activities and project development will be itemized regarding forecasting and evaluation, gaps, substitutability, institutions, and limitations.
3. Activity selection unit. The activity selection unit in the termly reviewed system determines guidelines for the future activities of each PHN. The individual care subsection includes accumulated daily reports and additional items that choose complexity, multiproblematic cases, and rapid changes in social needs. Meanwhile, the community-based activities and project development subsections comprise automatic checks based on accumulated daily reports and items involved in transitions between activity types, such as health crises or government policy changes.

## Discussion

### Principal Findings

This study will develop and validate an electronic recording and management system to evaluate the necessity of different public health nursing practices and accordingly consider how public health nursing practices may be improved; specifically, the system will focus on practices that fall into the categories of individual care, community-based activities, and project development. This study has several strengths. Validated items will evaluate the necessity of different public health nursing practices, suggest the best ones, and show the clinical reasoning of PHNs. The system will enable PHNs to record their daily activities and share them with their supervisors to reflect on and improve their assessment and the quality of care to promote health equity in community settings.

This study focuses on the links between individual care, community-based activities, and project development to assess the need for continued care. Most studies showed the framework and items of nursing assessment, interventions, and outcomes for individual patients [10-14]. By contrast, PHNs promote health equity and individual, family, and community health through direct care and policy development [15]. However, PHNs are often too busy with individual care to assess community health needs and implement population-wide activities [17]. By offering new insights into PHNs’ tacit knowledge, this study can also inform the best practices for them. Additionally, senior and managerial PHNs play a vital role in bridging community needs assessment and grassroots projects in ways that support novice PHNs. Clarifying and translating PHNs’ tacit knowledge into explicit knowledge would be essential to developing health care systems [35].

The result of this study will show the required time and burden for PHNs regarding each activity. Health care organizations required help ensuring high-quality care, owing to limited staff and other resources [36]. In this context, it is necessary to optimize human resources in health care. Identifying benchmarks for and measuring staff performance is crucial for effective human resource management and person-centered care [37]. Therefore, supervisory PHNs need to be able to identify the performance of each staff member and the effectiveness of their activities. Our system will support supervisory PHNs in promoting evidence-based human resource development.

Moreover, because the system requires PHNs to record their daily activities, it will enable PHN directors and staff to reflect on their practice periodically. Sharing the results of their daily activities with tier supervisors can help PHNs improve their performance and provide appropriate care for individuals, families, aggregates, and communities. Therefore, this study will improve the quality of care and address community health inequities.

### Limitations

This study has some limitations. First, due to the ongoing COVID-19 pandemic, we can only interview a few participants; additionally, we expect the response rate for our nationwide survey to be low. Meanwhile, it is also notable that our study participants will likely be health care workers interested in the study. While we will work to ensure that our sample will be nationally representative by asking all local governments in Japan to participate, this study will still be subject to this selection bias. We will be sure to carefully analyze and interpret the data accordingly.

### Conclusions

This exploratory sequential study will attempt to develop and validate an electronic recording and management system to evaluate the necessity of different public health nursing practices and determine best practices. The results will offer insights to develop a system that can help PHNs reflect on their daily routines and create benchmarks that can inform, thus improving on-the-job training by supervisory PHNs. This system will strengthen evidence-based public health nursing practices and management while improving the quality of care to enhance health equities in community settings.

## Abbreviations

ICT: information and communication technology
PHN: public health nurse

## Data Availability

The data set is not available to protect the participants’ privacy; moreover, participants did not provide written consent to share data.
